# The COSI trial: a study protocol for a multi-centre, randomised controlled trial to explore the clinical and cost-effectiveness of the Circle of Security-Parenting Intervention in community perinatal mental health services in England

**DOI:** 10.1186/s13063-023-07194-3

**Published:** 2023-03-14

**Authors:** Camilla Rosan, Kim Alyousefi-van Dijk, Zoe Darwin, Daphne Babalis, Victoria Cornelius, Rachel Phillips, Lani Richards, Hannah Wright, Steve Pilling, Pasco Fearon, Elena Pizzo, Peter Fonagy

**Affiliations:** 1 Department of Clinical, Educational and Health Psychology, University College London, London, UK; 2 Anna Freud National Centre for Children and Families, 4-8 Rodney St, London, N1 9JH UK; 3 University of Huddersfield, Huddersfield, UK; 4 Imperial Clinical Trials Unit, School of Public Health, Imperial College London, London, UK; 5 Department of Psychology, University of Cambridge, Cambridge, UK; 6 Department of Applied Health Research, University College London, London, UK

**Keywords:** Perinatal mental health, Circle of Security-Parenting, Parent-infant bonding, Child development, Child attachment, Randomised controlled trial

## Abstract

**Background:**

Perinatal mental health difficulties affect up to 27% of birthing parents during pregnancy and the first postnatal year, and if untreated are associated with difficulties in bonding and long-term adverse outcomes to children. There are large evidence gaps related to psychological treatment, particularly in group therapy approaches and parent-infant interventions. One intervention showing preliminary efficacious findings and user acceptability is Circle of Security-Parenting (COS-P), which is a brief, weekly, group programme. However, these studies were underpowered and predominantly non-randomised, and there has never been a research trial in England or with birthing parents experiencing severe and complex perinatal mental health difficulties. The aim of the research is to conduct a randomised control trial to test whether COS-P will reduce perinatal mental health symptoms in birthing parents accessing NHS perinatal mental health services, compared to treatment as usual (TAU). Secondary objectives include exploring whether the intervention improves parenting sensitivity, emotion regulation skills, attachment security and infant development. Additionally, the project aims to examine whether the intervention is acceptable to parents and NHS staff, and whether it is cost-effective.

**Methods:**

COSI is an individually randomised, single-blind parallel arm controlled trial with an embedded internal pilot aiming to recruit 369 participants in a 2:1 ratio (intervention: TAU). Participants will be recruited from ten NHS community perinatal mental health services in England and screened based on clinical levels of both mental health symptoms (average CORE-OM score ≥ 1.1) and postnatal bonding difficulties (total PBQ score ≥ 12). This trial has 90% power to detect a MCID of 5 points on the CORE-OM. Primary and secondary outcomes will be measured at baseline, 3, 7 and 12 months after baseline. Service use and quality of life measures will also be collected alongside a process evaluation of parents’ and interveners’ views and experiences.

**Discussion:**

This will be the first large pragmatic trial to test whether COS-P is effective for birthing parents with severe and complex perinatal mental health difficulties in improving their mental health symptoms. If shown to be effective, the intervention could be delivered widely across the NHS and other similar services globally.

**Trial registration:**

ISRCTN, ISRCTN18308962. Registered 18 February 2022.

**Supplementary Information:**

The online version contains supplementary material available at 10.1186/s13063-023-07194-3.

## Administrative information

Note: the numbers in curly brackets in this protocol refer to SPIRIT checklist item numbers. The order of the items has been modified to group similar items (see http://www.equator-network.org/reporting-guidelines/spirit-2013-statement-defining-standard-protocol-items-for-clinical-trials/).

**Table:** 

Title {1}	SPIRIT guidance: Descriptive title identifying the study design, population, interventions, and, if applicable, trial acronym. The COSI trial: a study protocol for a multi-centre, individually randomised controlled trial to explore the clinical and cost effectiveness of the Circle of Security-Parenting Intervention in community perinatal mental health services in England.
Trial registration {2a and 2b}.	SPIRIT guidance: Trial identifier and registry name. If not yet registered, name of intended registry. Item 2b is met if the register used for registration collects all items from the World Health Organization Trial Registration Data Set. Th ISRCTN, ISRCTN18308962. Registered 18 February 2022, https://www.isrctn.com/ISRCTNISRCTN18308962
Protocol version {3}	SPIRIT guidance: The current protocol version is 5.0, dated 08/12/2022.
Funding {4}	SPIRIT guidance: Sources and types of financial, material, and other support. This study is funded by the National Institute for Health Research (NIHR) Health Technology Assessment (HTA) Programme (NIHR131339) and is supported by the National Institute for Health and Care Research ARC North Thames. The views expressed are those of the author(s) and not necessarily those of the NIHR or the Department of Health and Social Care or any other organisations involved. The study/project is also supported by the NIHR Clinical Research Network (CPMS 50,730).
Author details {5a}	SPIRIT guidance: Affiliations of protocol contributors. ^1^ Department of Clinical, Educational and Health Psychology, University College London, London, United Kingdom ^2^ Anna Freud National Centre for Children and Families, London, United Kingdom ^3^ University of Huddersfield, Huddersfield, United Kingdom ^4^ Imperial Clinical Trials Unit, School of Public Health, Imperial College London, London, United Kingdom ^5^ Department of Applied Health Research, University College London, London, United Kingdom ^5^ Department of Psychology, University of Cambridge, Cambridge, United Kingdom
Name and contact information for the trial sponsor {5b}	SPIRIT guidance: Name and contact information for the trial sponsor. Trial sponsor: Anna Freud National Centre for Children and Families, 4–8 Rodney St, London N1 9JH, United Kingdom Trial partners: Imperial Clinical Trials Unit, School of Public Health, Imperial College London, London, United Kingdom University of Huddersfield, Huddersfield, United Kingdom Research Department of Clinical, Educational and Health Psychology, University College London, London, United Kingdom Institute of Epidemiology & Health, University College London, London, United Kingdom
Role of sponsor {5c}	SPIRIT guidance: Role of study sponsor and funders, if any, in study design; collection, management, analysis, and interpretation of data; writing of the report; and the decision to submit the report for publication, including whether they will have ultimate authority over any of these activities. The role of the Anna Freud Centre as sponsor is collecting all quantitative data, management of parts of that data, interpretation of data, writing of the report, and the decision to submit the report for publication, including ultimate authority over any of these activities. The Imperial Clinical Trials Unit is responsible for management of parts of the quantitative data, study design and statistical data analysis and reporting. The University of Huddersfield is responsible for collection, management, analysis and write up of the trial’s qualitative data. University College London is responsible for analysing the cost-effectiveness of the intervention, as well as providing expertise around adult and child mental health and outcome measures.

## Introduction

### Background and rationale {6a}

Mental health difficulties that develop, continue, or worsen during pregnancy and the first postnatal year (i.e. the perinatal period) are a significant public health concern globally. Perinatal mental health difficulties (PMHD) are common, with a population prevalence in birthing parents of up to 27% in high-income countries [1, 2] and higher rates being found in low- and middle-income countries [3]. They are also associated with marked morbidity and mortality for birthing parents, their infants, their wider family and society [4]. Untreated PMHD confers a significant financial burden; an economic evaluation based on UK data found that this amounted to £8.1 billion per birth cohort, with 72% of this cost attributed to the long-term morbidity of the child [5]. Although the development of this child morbidity is complex and multifactorial, research strongly indicates that it arises, in part, from changes in the quality of the parent-infant relationship when a birthing parent is experiencing mental health difficulties [6].

There is considerable international variation in how public services identify and manage PMHD, including within universal (e.g. maternity and child health) and specialist services. England has benefitted from rapid government investment in perinatal mental health services (PMHS) in the last decade, with more than £500 million [7, 8] of new funding being shared across the NHS to ensure that birthing parents with complex and severe PMHD can access evidence-based interventions in a timely manner. This programme of work has led to the establishment of multi-disciplinary NHS perinatal mental health services (PMHS) in every geographical region in England. PMHS need clinically effective and cost-effective psychological interventions. However, the evidence base for such interventions in the perinatal period is mixed with the most recent National Institute for Health and Care Excellence (NICE) guidance for antenatal and postnatal mental health identifying various gaps [9]. These include interventions that (a) work transdiagnostically with a range of (comorbid) perinatal mental health difficulties; (b) target both symptoms of psychopathology and parent-infant relationship quality; and (c) are delivered in a group format. Nonetheless, some group-based, transdiagnostic psychological interventions are showing preliminary efficacious findings and are already being adopted widely by psychologists working in NHS community PMHS. One of these interventions is Circle of Security-Parenting (COS-P) [10]. However, COS-P has not yet been rigorously evaluated in England and in the context of PMHS. The need for an English specific trial is particularly pertinent as many international trials that conclude an intervention outperforms TAU, does not do so in the England as NHS TAU is more comprehensive than many North American and even European service provisions (e.g. Family Nurse Partnership [11, 12]). We therefore propose to conduct a definitive trial of COS-P to determine its clinical and cost-effectiveness as an intervention offered to this population.

The proposed trial addresses the key gaps in the intervention evidence base discussed here: transdiagnostic approaches, bonding difficulties and group therapies. The rationale for the importance of each of these will be discussed in turn.

Birthing parents entering PMHS often present with multiple mental health difficulties and comorbidities [13], making it difficult for clinicians to select the most appropriate intervention for treatment, as NICE guidelines predominantly use a single diagnostic framework [9], e.g. high-intensity CBT for moderate to severe depression. However, there is growing evidence for transdiagnostic models of psychopathology, which suggest that many mental health difficulties are manifestations of a small number of core underlying features [14]. A body of research indicates that emotion regulation difficulties are one of these core transdiagnostic constructs [15, 16], which re-iterates the need for developing and testing treatments that target emotion regulation for individuals who present with multiple mental health difficulties. Emotion regulation is a particularly relevant intervention target in the postnatal period as it impacts both birthing parent and infant.

A key early developmental task is an infant’s acquisition of skills for regulating their emotional states [17]. Parents play a key role in helping with this, and in turn supporting the infant’s brain development [1]. There is strong evidence that perinatal mental health difficulties can disrupt this process. For example, it has been found that birthing parents with emotion regulation problems have difficulties thinking about their baby’s thoughts and feelings (i.e. reflective functioning; [18]), and are more likely to experience bonding problems. Therefore, research is needed to examine the effectiveness of treatments that address transdiagnostic constructs such as emotion regulation, particularly in PMHS. There has been very little research examining the effectiveness of interventions that target both perinatal mental health and bonding difficulties; particularly in relation to birthing parents of infants under 12 months, and critically with birthing parents with complex and severe perinatal mental health difficulties who are accessing PMHS. Where research has taken place with these populations, studies often have had very small sample sizes and poor-quality methodology and were conducted outside the UK, in the USA and Europe [19–23]. Furthermore, currently, PMHS assess and treat PMHD and bonding difficulties separately with different staff groups leading on the different difficulties [24]. It could therefore be cost-effective and potentially more acceptable to both parents and staff to deliver an intervention that addresses both needs.

A group context is likely to be particularly valuable for new parents with mental health difficulties, as the constructive, supportive environment created in the group as well as the commonality of the problems are known to diminish feelings of isolation, enable group members to share their mental health and parenting-related struggles, learn from each other and feel validated [25, 26].

A 2016 meta-analysis of studies involving COS-P programmes found a total of 10 eligible studies [19]; however, very few of these studies were RCTs, none of the studies included populations in the perinatal period and none of the studies specifically targeted samples with mental health symptoms. Since this review, four trials across Europe, Australia and the USA have evaluated the effectiveness of the COS-P intervention [20–23] with sample sizes of 141, 52, 221 and 72 respectively. Again, the conclusions that can be drawn from these studies are limited by being underpowered and again their lack of specificity to the perinatal period for complex and severe PMHD. A full-scale England-based trial is therefore warranted.

### Objectives {7}

The primary objective of this research is to conduct a randomised controlled trial (RCT) to determine whether COS-P reduces the mental health symptoms of birthing parents accessing specialist NHS community PMHS compared to Treatment As Usual (TAU).

The secondary objectives of this research are as follows:

- To explore whether COS-P improves emotion regulation difficulties, parental sensitivity, parent-infant bonding, attachment security and social support.
- To examine whether COS-P has an impact on infant development.
- To explore whether COS-P is acceptable and accessible to parents and NHS staff, e.g. exploring barriers and facilitators to taking part in COS-P, and exploring contextual factors that may influence the acceptability of COS-P.
- To explore possible mechanisms of change, e.g. exploring contextual factors that may influence effectiveness of COS-P.
- To determine whether COS-P is cost-effective or not.
- To investigate whether there is value of information associated with the trial results.

The primary hypothesis is that COS-P is superior to TAU with respect to decreasing mental health difficulties in birthing parents, measured with the CORE-OM averaged over the 3-, 7- and 12-month follow-up assessments. Secondly, it is hypothesised that relative to the control group, participants receiving COS-P will show an increase in parental emotion regulation, increase in quality of life, increase in social support, increase in parenting sensitivity, decrease in parent-infant relationship difficulties, increase in infant attachment security and an improvement in infant global and socio-emotional developmental outcomes.

The emphasis of the qualitative process evaluation concerns acceptability and accessibility; however, it is recognised in the Medical Research Council guidance that additional questions may be identified during the evaluation, requiring that the process evaluation ‘be designed with sufficient flexibility and resources to allow important emerging questions to be addressed’ [27].

### Trial design {8}

This is a multi-centre RCT with follow-up assessments for up to 1-year post-baseline. Programme theory informed all decisions on trial design and analyses plans. The trial includes a 2:1 randomisation ratio with all outcome assessors blind to intervention allocation. Participants are randomised to either:

1. The intervention arm consisting of COS-P plus TAU in a PMHS
2. The control arm consisting of TAU in a PMH

The COSI trial uses a mixed methods design (see Additional File 1). All parent participants are invited to one pre-intervention and three post-intervention assessments with quantitative measures. Additionally, the trial includes an embedded process evaluation, in which a combination of survey methods, interviews and focus groups will be used to gather the views and experiences of parent participants in the intervention arm, and clinical staff involved in delivering the intervention. Consistent with the Medical Research Council guidance on developing and evaluating complex intervention [28], the process evaluation will enable exploration of the acceptability and accessibility of COS-P with different groups of stakeholders and exploration of contextual factors that may influence mechanisms of change and the effectiveness of COS-P, which can be used to refine the trial’s Theory of Change model.

The trial includes a 12-month internal pilot to establish that trial procedures work well in terms of recruiting and retaining participants. Additionally, intervention attendance and fidelity to the manual of COS-P delivery will be checked. The internal pilot progression criteria and further details are listed in Additional File 2. In the event of any of the criteria not being met, the trial team will work together with the Trial Steering Committee (TSC) and Data Monitoring and Ethics Committee (DMEC) to review strategies for improvement or the TSC will recommend the termination of the trial.

## Methods: participants, interventions and outcomes

### Study setting {9}

This trial aims to recruit 369 parents accessing specialist NHS community PMHS in England for the treatment of moderate to severe PMHD. Additionally, the trial will aim to recruit around 20 staff participants from associated PMHS who are involved in delivering the intervention. An exhaustive list of associated PMHS can be found in the online full trial protocol (https://fundingawards.nihr.ac.uk/award/NIHR131339).

### Eligibility criteria {10}

#### Parent participants

Inclusion criteria for birthing women and other birthing parents (referred to collectively as ‘birthing parents’ throughout this paper).

- Are accessing an NHS community PMHS from one of the recruiting sites.
- Have a child aged 0–12 months with no severe illness or developmental disorder.
- Score 1.1 or more as their average score on the Clinical Outcomes in Routine Evaluation-10 (CORE-10; [29]) or score 1 or more as their average score on the Clinical Outcomes in Routine Evaluation-Outcome Measure (CORE-OM; [30]).
- Score 12 or more on the Postpartum Bonding Questionnaire (PBQ; [31]).
- Are aged at least 18 and are willing and able to give informed consent.
- Are able to attend groups without being under the influence of substances.

Exclusion criteria for the trial are birthing parents who:

- Do not have a minimum of conversational English.
- Have received COS-P previously.
- Are experiencing active psychosis.

#### Staff participants

Staff participants will include staff members who are trained COS-P interveners and involved in the delivery or supervision of the groups.

#### Recruitment and intervention sites

PMHS were selected based on the following criteria: (1) willingness and capacity to participate in the trial; (2) an absence of COS-P in their standard care; (3) an absence of COS-P delivery in the geographical area they cover; (4) availability of at least one practitioner psychologist or similar member of staff willing to be trained in COS-P. The inclusion criteria to become facilitators are that the staff participants can provide the time commitment associated with the COS-P training and group delivery within their current role, and their being experienced in delivering group therapy sessions and / or parent-infant work.

### Who will take informed consent? {26a}

#### Birthing parents—quantitative data collection

Trial recruitment takes place across seven, 4-week recruitment blocks over the 20-month recruitment period. Information about the trial will be publicised widely among clinical colleagues working in all recruiting PMHS sites. PMHS staff will approach birthing parents about the trial during standard screening meetings (e.g. intake assessments, review meetings). Participants that meet the CORE-10/CORE-OM and PBQ eligibility criteria (further details on screening can be found in Additional File 3) will be given a Participant Information Sheet (PIS) and recruitment leaflet and asked to provide verbal consent to be contacted by a member of the research team to discuss this information further. This will be done with the help of an interpreter if needed. Existing scores on the CORE-10 or CORE-OM and the PBQ assessed within 6 weeks prior to the beginning of a recruitment block are also accepted as screening scores for the trial. Staff members completing screening measures will not be involved in the intervention delivery; however, it is probable that they will be involved in the delivery of TAU at some sites. Birthing parents who do not provide verbal consent to be contacted by the trial team will be asked to complete a short survey about their decision. The survey was created in collaboration with the trial’s Expert by Experience (EbE) panel and includes factors/barriers which may have influenced the birthing parents’ decision as well as an option to list additional reasons. Contact details and CORE and PBQ scores of those who do provide verbal consent will be shared securely with the research team via email. A member of the study team then contacts the individual to discuss the trial in detail, assess eligibility and obtain informed consent. In all instances, potential participants will have at least 24 h before deciding whether they wish to take part. All trial documents, including the PIS, will be provided in alternative languages where required. Informed consent for the interview will be recorded using Microsoft Forms. A copy of the PIS and completed informed consent form (ICF) will be shared with participants and their clinical team. The family doctor of each participant will be notified of their participation.

#### Birthing parents—qualitative data collection

For the purpose of the qualitative component of the trial, birthing parents assigned to COS-P will be categorised as follows to ensure the relevance of questions asked: *non-starters* (who do not begin the group), *non-completers* (who begin the group but do not receive what is considered a clinical dose, indicated by attending fewer than six sessions) and *completers* (who begin the group and attend at least six sessions). Approximately 3 months after the baseline assessment has been completed, all birthing parents in the intervention arm will be asked to share their views and experiences regarding COS-P, including barriers and facilitators to taking part. Non-starters will be asked to complete a three-item ‘non-starters’ survey to report the reasons. Completers and non-completers will be asked to complete a short experience survey and indicate their interest in taking part in an interview. All surveys can be self-completed or completed with the assistance of a member of the study team, either remotely (by telephone or Teams) or in person. A subsample of completers and all non-completers will be invited by a member of the study team to be interviewed and provided with a PIS and opportunity to ask any questions. Where necessary, the PIS will be translated into alternative languages and interpreter support will be provided to ensure informed consent. Informed consent for the interview will be recorded using Microsoft Forms. A copy of the completed ICF and PIS will be shared with the participant for their records.

#### Staff participants—focus groups

The research team provides a PIS and ICF to all staff participants who facilitate or supervise delivery of the group, to gain their informed consent for the focus group, or for an interview in circumstances where they leave their role before the focus groups take place.

#### Staff participants—fidelity coding

An information sheet will be shared and informed consent will be collected by the research team for the staff members’ third COS-P group to be video recorded.

### Additional consent provisions for collection and use of participant data and biological specimens {26b}

Not applicable, this trial does not collect biological specimens.

## Interventions

### Explanation for the choice of comparators {6b}

Participants allocated to the TAU arm of the trial will receive the usual support offered by their service. TAU at each PMHS in the trial is defined by a national service specification [32]. We will collect participant reports of TAU at data collection assessments via a standardised measure: the Client Service Receipt Inventory (CSRI), supplemented with PMHS report of interventions offered by their service.

### Intervention description {11a}

All participants allocated to the intervention arm of the trial will receive COS-P alongside the TAU offered by their service. COS-P is a group intervention designed to provide social support and peer connection in parents experiencing bonding difficulties with their child. Based on psycho-educational, cognitive-behavioural and psychodynamic theories and techniques, COS-P is delivered by a trained, supervised, NHS staff member (predominantly doctorate level clinical and counselling psychologists) to groups of 4–6 birthing parents. The intervention involves 8 treatment modules which are delivered remotely online over 10 weekly, 90-min sessions (see Table 1 for details). The module contents include video clips of parent–child interactions and reflections of previous COS-P participants. Topics covered in COS-P include the basic concepts of attachment, responding to children’s affective states, reflecting on caregiving struggles and noticing mean (hostile), weak (helpless) and gone (neglecting) parenting. Where possible, the first session and one additional session are delivered in a face-to-face format, at local, accessible venues. All remaining sessions will be delivered virtually, via Microsoft Teams. Interpreters may join group sessions to support participants where required.

**Table 1:** Summary of COS-P intervention modules schedule

Group session	Manual modules and corresponding themes
1	1 (Welcome to Circle of Security-Parenting)
2	2 (Exploring Our Children’s Needs All The Way Around the Circle)
3	3 (‘Being With’ on the Circle) 4 (‘Being With’ Infants on the Circle) 5 (The Path to Security) 6 (Exploring Our Struggles) 7 (Rupture and Repair in Relationships)
4	
5	
6	
7	
8	
9	
10	8 (Summary and Celebration)

The intellectual property (IP) for COS-P is held by COS International. No restrictions exist on the right to use the materials of the COS-P intervention, and no costs are associated with its use from the creators or their organisation, other than the costs to train in the intervention. The COSI trial examines COS-P in a perinatal version that is delivered to birthing parents with perinatal mental health difficulties. COS International are aware of the COSI trial and are involved to provide consultation and fidelity coaching to all the trial interveners. Any foreground IP relating to COS-P (e.g. the perinatal adaption of the intervention) lies with COS International. Any foreground IP relating to scientific results of the trial is co-owned by the sponsor and partners.

### Criteria for discontinuing or modifying allocated interventions {11b}

Discontinuation of the allocated intervention is possible after a participant withdraws from the intervention and/or trial or based on clinical judgement by the intervention facilitator, e.g. when a participant’s mental health deteriorates and is not advised by their clinician to continue.

### Strategies to improve adherence to interventions {11c}

Adherence to the intervention is facilitated in several ways. Firstly, potential participants are informed of the dates, times and location of the intervention sessions before they decide to participate. Potential issues around attendance are discussed and resolved, i.e. the trial covers childcare and transportation costs for the face-to-face sessions and a tablet with data can be arranged for the virtual sessions. Facilitators are encouraged to schedule pre-group individual calls with everyone assigned to their group and to reach out to anyone who misses a session to allow a smooth re-entry in the group.

Also, the trial explores treatment fidelity of COS-P within the trial. Each facilitator delivering COS-P within the trial will be trained in its delivery by accredited COS International trainers and are given a clear group delivery manual to follow. Additionally, all facilitators undertake 20 h of fidelity coaching. The fidelity coaching includes 10, 2-h coaching sessions with a supervisor from COS International. Lastly, all facilitators will receive supervision from expert therapists at their PMHS trained in parenting interventions and/or COS-P.

Fidelity of delivery will be checked by recording all ten sessions of one full group for each intervener. A randomly selected 20% of recorded videos will be coded for fidelity to the manual. A criterion of 75% adherence to the COS-P manual will be used to ensure that the treatment administered meets the standards of fidelity required for the trial.

Additionally, attendance to the intervention sessions is monitored and 6/10 sessions is considered a clinical dose. When participants miss a session, an individual catch-up session is offered to help them re-enter the group and this does not itself constitute attendance of an intervention session.

### Relevant concomitant care permitted or prohibited during the trial {11d}

All participants receive TAU from their PMHS and COS-P cannot be offered to participants in the control arm.

### Provisions for post-trial care {30}

No provisions for post-trial care are in place. Additionally, there is no anticipated harm and compensation relating to harm for trial participation.

### Outcomes {12}

#### Outcome measures—quantitative data collection

The primary outcome measure is the Clinical Outcomes in Routine Evaluation – Outcome Measure (CORE-OM; [33]), included in all trial assessments. The CORE-OM is a 34-item measure of psychological distress and is one of the most widely used outcome measures in secondary care mental health services.

Secondary outcome measures exploring parent-infant bonding, experienced childhood maltreatment, emotion regulation, parenting quality, health-related quality of life, health service use, child development and attachment style, adverse events, and COS-P cost-effectiveness will also be completed throughout the trial. Details on these measures are provided in Additional File 4.

#### Outcome measures—qualitative data collection

The qualitative data will explore acceptability and accessibility of COS-P and possible mechanisms of change, from the perspectives of parent participants in the intervention arm and NHS staff who are involved in the delivery of COS-P within the trial. This will be achieved through surveys, interviews and focus groups as outlined later.

### Participant timeline {13}

Please see Additional File 1 for details relating to the participant timeline.

### Sample size {14}

A change in CORE-OM of 5 has been proposed as a meaningful improvement and a reliable change that exceeds that which might be expected by chance [9]. In a 2009 trial, Morrell et al. [34] reported that in a sample of birthing parents with Edinburgh Postnatal Depression Scale (EPDS) scores ≥ 12 (i.e. birthing parents with probable perinatal mental health difficulties), the mean score on the CORE-OM had a standard deviation of 0.5. A change in total score of 5 equates to an average mean item change of 0.147. The CORE-OM total change score we are using was strongly endorsed by our EbE panel as being meaningful. A between measurements within subject correlation of 0.35 were estimated, from unpublished clinical audit data on 71 birthing parents in a specialist community perinatal mental health service.

We choose to use a 2:1 ratio to randomise to increase motivation of participants to take part in the trial due to the perceived benefit of the intervention, and with consideration to the practicality of filling up recruitment blocks in a timely manner, as we need at least 4 participants within each COS-P group.

With 104 parents in the control arm and 208 parents (*N* = 312) in the intervention arm, we will have 90% power to detect a minimally clinically important average mean item change of 0.147, assuming a *SD* of 0.5, three repeated measurements with a correlation of 0.35 and using a 5% significance threshold. Previous small trials have reported 15–20% missing at follow-up [20, 21] by the end of the trial. In the current trial, participants will be rigorously followed up and those who have at least one post-randomisation measurement will be included in the analysis. We therefore assume it is reasonable to obtain one post-randomisation measurement of the primary outcome in at least 90% and therefore factor in 10% missing. As the intervention is delivered in groups, there is potential for clustering of the outcomes in the active arm. We do not have reliable data to inform us what the intra-cluster correlation (ICC) coefficient in this context is. However, even if the ICC were large (> 0.05), the intervention group size is small so the effect will be limited. It is not expected this clustering will greatly affect the results. To protect against any potential effect in the absence of information, we inflated the sample size by an additional 5% to take into account the potential for one-arm clustering. Taking into account the potential for clustering (5%) and missing data (10%), we will aim to recruit a total of 369 birthing parents (*n* = 246 and *n* = 123 per arm). This was calculated using the time-averaged difference test in the PASS statistical software.

For the embedded qualitative component, we have estimated interviewing 20–30 completers, which is consistent with other HTA-funded process evaluations (e.g. ISRCTN12655391, ISRCTN34701576) and aim to interview all non-completers. The total sample size will be guided by principles of data saturation concerning meaning [35] and with emphasis on data quality. It is therefore not possible to determine the exact sample size in advance. Focus groups with staff will use total population sampling, i.e. inviting all members of those groups.

### Recruitment {15}

We aim to recruit a total of 369 birthing parents in up to seven, 4-week recruitment blocks in each of the sites over a 20-month period. Based on this, we have calculated that we will need to screen 1262 birthing parents during this time, which draws from some of the most conservative data in previous trials and published studies on recruitment (65% of birthing parents consenting to be screened, 75% meeting the screening criteria and 60% consenting to be randomised across the study period) [20, 21, 36].

## Assignment of interventions: allocation

### Sequence generation {16a}

At the end of each recruitment block, participants will be randomly allocated to one arm of the trial (2:1, intervention:TAU stratification) using a web-based randomisation system using computer-generated random list stratified by trial site and recruitment cohort. All researchers collecting baseline and follow-up data, as well as assessors of outcome measures, will be blinded to group allocation.

### Concealment mechanism {16b}

The randomization list is concealed using an online web-based randomisation system integrated into the Electronic Data Capture. Participants are entered into the system and consented prior to allocation being revealed.

### Implementation {16c}

The allocation sequence is generated by the trial statistician. Participants are enrolled by the study team and assigned to the intervention by the trial manager using the EDC system.

## Assignment of interventions: blinding

### Who will be blinded {17a}

Study team members collecting quantitative data and study quantitative outcome assessors are blinded to the intervention. It is not possible to blind the analysts due to the 2:1 allocation ratio.

### Procedure for unblinding if needed {17b}

If study team members are unblinded, this will be recorded via a protocol deviation form within the trial database. The unblinded team member will complete no further data collection or outcome assessments with that participant for the duration of the trial. As participants are not blinded to allocation, there is no requirement to have a facility available for unblinding participants.

## Data collection and management

### Plans for assessment and collection of outcomes {18a}

#### Data collection and management—quantitative data collection

Participants are offered the possibility of face-to-face (e.g. at home) and virtual trial assessments (via Microsoft Teams) at baseline, 3-month and 7-month follow-up assessments. Baseline trial assessments take place prior to randomisation and the first COS-P session. The trial aims to have no more than 4 weeks between randomisation and the first COS-P group session. As the 12-month follow-up assessment includes the Strange Situation Procedure (SSP; [37]) used for assessing attachment security of the child, this trial assessment takes place in person at a venue local to the participant. Please see Table 2 for further details regarding the data collection schedule. Apart from the video-recorded parent–child interaction and the SSP, participants are given the choice to self-complete the measures online, or to complete them together with a member of the research team. Details on all study measures are provided in Additional File 4.

**Table 2:** COSI study data collection schedule

	**Screening**	**Baseline**	**COS-P group (intervention arm only)**	**3-month f/u**	**7-month f/u**	**12-month f/u**
Informed consent	X	X				
CORE-10	X					
Inclusion and exclusion criteria		X				
Demographics		X				
Randomisation		X				
CORE-OM		X		X	X	X
PBQ	X	X		X	X	X
CTQ-SF		X				
DERS		X		X	X	X
ASQ-SE		X		X	X	X
ASQ-3		X		X	X	X
Sensitivity scales		X		X	X	X
CSRI		X		X	X	X
EQ-5D-5L		X		X	X	X
CORE-6D		X		X	X	X
SSP						X
Adverse events		X		X	X	X
Short experience survey			X			
Qualitative interviews			X			

### Data collection and management—qualitative data collection

Qualitative data collection is completed shortly after the 3-month follow-up of the trial. All participants in the intervention arm of the trial are asked to share their views and experiences using survey materials designed for the trial in collaboration with the EbE panel; to explore the acceptability and accessibility of COS-P, see Additional File 5. The survey will additionally provide a sampling framework for the interviews with completers (i.e. those who attend at least six sessions), using maximum variation sampling [38] to ensure that parents with a wide range of background characteristics are included (e.g. diversity in relation to ethnicity, relationship status, age, parity, infant age) and from across sites and recruitment blocks. All non-completers (i.e. those who begin COS-P but attend fewer than six sessions) are invited to be interviewed.

Interviews are approximately 1 h long and follow a topic guide based on the trial aims (see Additional File 6). In addition to this, focus groups with NHS Staff members involved in the delivery of COS-P within the trial are completed. These focus groups explore staff views of the intervention and experiences of delivery, either through direct facilitation or supervision of staff involved in facilitation. Focus groups are 1.5–2 h long and follow a pre-determined topic guide (see Additional File 7). Any facilitators who leave the trial prior to a focus group having taken place will be offered individual interview. Both the interviews and focus groups will be audio/audio-visually recorded and transcribed verbatim.

### Plans to promote participant retention and complete follow-up {18b}

It is intended that newsletters regarding the trial will be shared with participants in the study to promote engagement. Parent participants also receive a £10 voucher per trial assessment as reimbursement for their time. An additional £20 voucher will be provided as reimbursement for parent participants who take part in an interview for the qualitative assessment.

If a participant chooses to withdraw from all study procedures, no further outcome data will be collected. Any information regarding the reason for the withdrawal will be recorded in the trial database. If a participant chooses to withdraw from the study intervention only, all future follow-up visits will be completed as per the study protocol.

### Data management {19}

#### Data collected on the EDC system

Study outcome measures are entered directly into the REDCap EDC system [39, 40] by participants (online, using a unique access code) or research staff with role-based and password-protected access, who have undertaken the necessary EDC training. The database has passed validation and User Acceptability Testing. Range checks for data values are built into the system. Coded outcomes of observational measures (i.e. sensitivity scales, attachment style and facilitator fidelity to the manual) are only entered into the system by research staff. COS-P report forms are completed by PMHS staff involved in delivery, and entered onto the REDCap system by research staff. PMHS staff have no access to the REDCap system. Monitoring and Source Data Verification will be performed electronically by the Trial Manager or designee as defined in the study monitoring plan. This applies to the COS-P report forms filled out by PMHS staff as well as coding outcomes recorded separately (i.e. sensitivity scales, attachment style and facilitator fidelity to the manual). Once the data are declared clean, i.e. no outstanding queries or issues, and locked by the Study Manager at the end of the study, the CI must complete the signature panel associated with each subject. The Study Manager will request CI approval and then proceed with Database Lock (DBL) and final Data Extract request.

#### Data outside the EDC system

Video recordings of participants and their children (needed to code the sensitivity scales and attachment style) are recorded and stored by research staff outside of the EDC system. Video recordings of intervention sessions are recorded by clinical staff in PMHS and then shared with, and stored by, the research staff at the sponsor. Video recordings of intervention sessions are deleted within 1 year, or when coded if earlier than 1 year. All other video materials and outcome measures are securely archived for 10 years.

#### Qualitative data

Survey data, recordings and transcripts of interviews and focus groups are stored securely online in the UK. Any segments of recording that are unclear will be time stamped by the transcribers, and the research team will check the original recording for accuracy. Recordings will be destroyed upon the study’s completion. Anonymised transcripts and analysis files will be stored securely for 10 years after the study’s completion.

### Confidentiality {27}

Prior to consent, the participant’s name and contact details are shared with the research team by their PMHS once verbal consent to do so has been received. All further personal information is self-reported by participants once included in the trial. All identifiable information will be stored on an encrypted server at the Anna Freud Centre, separate from all outcome data collected. Each participant will be allocated a unique trial ID, and all questionnaire data will be stored in the database according to this trial ID. No identifiable information will be stored in the trial database. Qualitative data will be linked to the main dataset via this unique trial ID number.

Parent participants are informed that all assurances on confidentiality will be strictly adhered to, unless a safeguarding issue regarding potential harm or danger to themselves or another individual becomes apparent. In this case, the concern would need to be referred to the Trial Manager and the relevant healthcare professional. If any risk to the child’s development is identified during the study, the Trial Manager will inform the parent participant and the relevant healthcare professional for further discussion.

### Plans for collection, laboratory evaluation and storage of biological specimens for genetic or molecular analysis in this trial/future use {33}

Not applicable as no biological samples are to be collected.

## Statistical methods

### Statistical methods for primary and secondary outcomes {20a}

The primary analysis aims to estimate the treatment policy estimand. We will use the intention-to-treat principle including all participants who undergo randomisation and have at least one post-randomisation measure at the 3-month, 7-month or 12-month follow-up trial assessment. As a result of this, the number of participants missing from the primary analysis model is expected to be low. A safety population consisting of all participants who attend at least one session of the assigned intervention will be used for the analysis of adverse events. For participants in the TAU arm of the trial, this will be defined as any participant randomised to TAU as per the intention-to-treat population. Baseline characteristics will be summarised by treatment arm and by using suitable measures of central tendencies. The flow of participants through the trial and trial results will be reported according to Consolidated Standards of Reporting Trials (CONSORT).

#### Primary analyses

For the primary analysis, we will use a mixed effects linear regression model to estimate the mean difference in CORE-OM between arms over 3-, 7- and 12-month follow-up trial assessments and a Bayesian mixed effects linear regression model using vague priors. From the Bayesian model, we will obtain the posterior probability of the intervention being superior to TAU, as well as the posterior probability of treatment effect exceeding the pre-specified Minimal Clinically Important Difference (MCID) of 5 points in the CORE-OM. In the mixed effects linear regression model, participants and recruitment cohort (in the intervention arm to account for group clustering in one arm) will be included as random intercepts. Fixed effects in the model will be intervention arm, site, and baseline CORE-OM, infant sex, infant age and infant first born status [36]. The mean difference in CORE-OM over 3-, 7- and 12-month follow-up trial assessments will be reported, with accompanying 95% confidence intervals, and *p*-value will be presented.

A Bayesian mixed effects linear regression model will be fitted and follows the same form as the frequentist mixed effects linear regression model described above. We will use vague (large variance) normal priors for regression coefficients and inverse-gamma priors for the error variance and for the variance of random intercepts which have been chosen to be uninformative. Model convergence will be investigated for the parameters of primary interest (specifically the treatment effect estimate) using graphical diagnostics. The intervention effect will also be estimated at the 3-, 7- and 12-month follow-up trial assessments using a Bayesian mixed effects linear regression model with a model including time point and adding a time-by-intervention arm interaction.

#### Sensitivity analysis on primary

The analysis using mixed effects linear regression model will be valid under a Missing At Random (MAR) assumption. If the proportion of participants that have no post-randomisation measures is above 5%, we will conduct an additional analysis using controlled multiple imputation to examine the impact of Missing Not At Random (MNAR).

#### Mechanism of action on primary

If the primary analysis indicates a treatment effect, then we will undertake a mediation analysis to explore the mechanisms underlying the intervention using a structural equation modelling approach. Variables to be included as potential mediators include parental sensitivity (as measured by the NIHD Sensitivity Scales), emotion regulation (as measured by the DERS) and life changes (e.g. the start of social care for the family) and relationship status (as measured by the demographic questionnaire and CSRI).

Pre-specified subgroup analysis will be performed for the primary outcome to explore the uniformity of the treatment effect by adding a treatment-by-subgroup interaction term to the primary analysis model (or test for trend where appropriate) for the following:

- History of mental health difficulties
- Experienced childhood maltreatment
- Geographical area
- Age
- Ethnicity
- Deprivation (as measured by personal gross yearly income)
- Relationship status

#### Supplementary analyses on primary

We will also undertake supplementary analyses to estimate the intervention effect in those that received the intervention sessions as planned. This is undertaken using a counterfactual approach where we will initially define a ‘complier’ (Y/N) as an individual who attends at least 60% (i.e. 6 of the 10) intervention sessions. We will also examine alternative definitions of a ‘completer’ estimating the effect of attending an increasing number of sessions [1–10].

#### Secondary analyses

Analysis of the secondary efficacy outcomes will be undertaken following the same framework as the primary outcome model with a time-by-intervention interaction using appropriate generalised linear models. For each continuous outcomes including the DERS, PBQ, ASQ-3, ASQ-SE and the NICHD Sensitivity Scales, a mixed effects linear regression model will be fitted as described above for the primary outcome. Trajectories of the predicted estimates with accompanying 95% confidence intervals from the mixed effects models over time will be displayed graphically.

Any secondary binary outcomes will be analysed using a generalised linear model fitted with a binomial distribution and logit link function and treatment effects are reported as odds ratios with 95% confidence intervals. Any secondary categorical outcomes will be analysed using a generalised linear model fitted with a binomial distribution and ologit link function for ordered categorical responses and the mlogit link function for unordered categorical responses and treatment effects reported as odds ratios (ologit model) or relative risk ratios (mlogit model) with 95% confidence intervals.

For the analysis of any time-to-event outcomes, treatment effects will be modelled using a proportional hazards time-to-event model. Kaplan–Meier estimates will also be plotted with confidence intervals for each treatment arm with extended at-risk tables [41].

##### Adverse events

Kaplan–Meier plots are used to examine rates of withdrawals by arm due to any AE. The number of participants requiring social care involvement for the family will be tabulated by arm and Kaplan–Meier plots will be used to examine the time to social care involvement by arm. All AEs are tabulated by arm and severity for the number of participants with at least one adverse event and the number of adverse events. We also calculate odds ratios and incident rate ratios and their 95% CIs for binary and count AE outcomes at SOC level using logistic regression and Zero-Inflated Poisson model or negative Binomial model, following the same framework as the primary analysis model using appropriate generalised linear models with adjustments. The results from these models are then presented graphically along with the raw counts using visual approaches such as the dot plot [42].

A detailed statistical analysis plan will be written prior to data lock and will detail all analysis models and model checks to be performed.

#### Qualitative data analysis

Survey data response rates and descriptives on the demographics of respondents will be summarised in order to describe the sample of respondents. These data and responses to closed questions will be subject to basic descriptive statistics including frequency counts and cross-tabulation. Responses to open-ended survey questions will be analysed using content analysis which involves generating descriptive codes summarising text responses and counting the frequency of those codes within the dataset.

The qualitative data collected during focus groups and interviews regarding parent and staff experiences will be transcribed verbatim by a confidentiality-bound professional transcription service. The data will be managed using NVivo and analysed using the Braun and Clarke thematic analysis approach [43]. Initially, a selection of the transcripts will be independently coded line-by-line by a qualitative sub-team to generate initial codes and search for candidate themes. These will then be reviewed and refined in a face-to-face meeting before undertaking further coding of subsequent transcripts. To promote rigour, peer debriefing will be used, with the researchers scrutinising each other’s interpretations and searching for disconfirming evidence. The emerging themes will be discussed with the EbE panel to ensure credibility and relevance for service users. We will explore alternative interpretations by revisiting transcripts, and refining the analysis supported by a series of remote and face-to-face discussions, until a satisfactory analysis is reached with agreement of final themes. Anonymised quotations will be used to illustrate the themes and a detailed audit trail will be recorded, summarising the development of themes.

#### Economic analysis

We will perform a within-trial economic evaluation comparing the costs and outcomes of COS-P versus TAU. We will assess the costs of implementing and delivering the intervention (e.g. cost of each session, including video projection, practitioner psychologist time) and the cost of TAU. We identify and measure health care resource use (e.g. GP consultations, psychological consultation, medications) through the CSRI [44] and using standard unit costs. The analysis will be performed by adopting the perspective of the UK NHS and Personal Social Services. Outcomes are measured using the EQ-5D-5L questionnaire [45] (but we will also explore translating the CORE-OM [33], and CORE-6D [46] into utility) to generate QALYs for the 12-month follow-up. The economic evaluation estimates the incremental cost per QALY associated with COS-P. Net monetary benefit of the intervention and TAU is assessed using the NICE lower and upper threshold [47]. If there is a significant outcome effect, a decision analytic model will be used to extrapolate the results over the longer term. Sensitivity analysis will be performed to control for uncertainty in the parameters and data.

### Interim analyses {21b}

An internal pilot will be embedded in the trial to assess recruitment rate by site, adherence to the intervention, fidelity to intervention, time to starting the intervention in the intervention arm, number and type of ‘treatment as usual’ received in control and intervention arm and overall trial retention. This information will be reviewed 12 months after recruitment has started. The review of adherence to the intervention and fidelity to intervention will be undertaken by the DMEC and the review of recruitment and retention will be undertaken by the TSC, both consisting of largely independent experts across the fields of mental health, parenting research, health economics, trial statistics and public involvement. We will use a traffic light system and the stop/go criterial can be found in Additional File 2.

### Methods for additional analyses (e.g. subgroup analyses) {20b}

There will be no additional analyses.

### Methods in analysis to handle protocol non-adherence and any statistical methods to handle missing data {20c}

As we target the treatment policy estimand, we will include all participants in the analysis including those that did not adhere to the protocol. A supplementary analysis will be performed using a counterfactual approach to estimate the treatment effect in those that adhered to the treatment protocol. Participants who have missing data points will be included in the analysis under a Missing at Random assumption implicit when using the longitudinal model. If there are more than 5% of participants who withdraw completely from the study or do not complete at least one post-baseline measurement, then we will undertake Multiple Imputations, again making a Missing at Random assumption. We will conduct an additional (sensitivity) analysis using controlled multiple imputation to examine the impact in case of Missing Not At Random (MNAR).

### Plans to give access to the full protocol, participant-level data and statistical code {31c}

The full protocol is made available on the funder’s website. The study team will retain the exclusive use of data until publication of all planned primary and secondary analyses have been completed. Following this, the anonymised quantitative dataset and extracts of the statistical code will be available from the corresponding author on reasonable request.

## Oversight and monitoring

### Composition of the coordinating centre and trial steering committee {5d}

The research team involved in the day-to-day delivery of the trial meets on a weekly basis, while the wider study team meets monthly, to discuss any current issues and updates. The Trial Management Group, including all co-applicants, meet every quarter. Additionally, the trial is supported by a Patient and Public Involvement co-applicant who leads an active Expert by Experience (EbE) panel. The EbE panel consists of around ten panel members and its role is to ensure that the participants’ perspective is kept in mind in every aspect of the COSI Study. The panel meet quarterly to provide feedback on participant facing documents as well as provide useful insights in aiding the smooth operation for the research team and facilitators. Individually, the panel members’ involvement can vary from giving feedback on certain documents/scenarios to database testing that ensures the systems we have in place are user friendly and that the research assistants are equipped to smoothly run study visits. Panel members are also invited to the COSI monthly meetings, where they can share live feedback with the wider research team. The panel also has two subgroups in which they focus on specific aspects on the study. There is the EDI subgroup, who look to ensure that the study is being as inclusive as possible when recruiting parents who are ethnically diverse. They meet on a semi-regular basis to discuss ways in which we can make this study more accessible to them, and things we should take into consideration when communicating with marginalised communities. The other subgroup supports the qualitative aspect of the study. This subgroup meets at four points during the study, to code and share findings from participants during interviews and from facilitators in focus groups.

The Trial Steering Committee (TSC) has been convened to oversee the progress and conduct of the trial, including a selection of the internal pilot criteria. Membership of the TSC includes an independent chair, independent statistician, independent PPI member, independent experts including experts by experience, and representatives of the study team. The TSC met at the beginning of the trial and will meet annually at a minimum for the trial’s duration.

### Composition of the data monitoring committee, its role and reporting structure {21a}

The Data Monitoring and Ethics Committee (DMEC) is fully independent and responsible for overseeing the safety of the trial and a selection of the internal pilot criteria. The DMEC met at the start of the trial and will meet annually at a minimum with meetings taking place prior to the TSC in order to facilitate reporting from the DMEC to the TSC.

### Adverse event reporting and harms {22}

At each follow-up data collection time point, participants are invited to complete a short survey regarding solicited adverse events (both physical and social) experienced during the trial. Adverse events which are not considered to be related to the trial intervention or procedures and are not one of the solicited adverse events detailed in this questionnaire will not be recorded in this way. However, unsolicited adverse events may be reported directly by participants during data collection visits or by the participant’s PMHS. All related adverse and serious events will be reported to the Chief Investigators, TSC and DMEC. In the event of a serious adverse event (SAE) occurring during the subject’s participation in the study, the SAE must be reported to the CI and the Sponsor.

All related and unexpected SAEs will be notified to the Research Ethics Committee (REC) and the Sponsor within 15 days of the Chief Investigator becoming aware of the event. Follow-up of participants who have experienced a related or unexpected SAE will continue until recovery is complete or the condition has stabilised.

Annual safety reporting will be included in the Annual Progress reports submitted to the Sponsor and the Research Ethics Committee, on the anniversary of Ethics approval each year. The Annual Progress Report will detail all SAEs recorded.

If any urgent safety measures are taken, the Chief Investigator/Sponsor shall immediately, and in any event no later than 3 days from the date the measures are taken, give written notice to the relevant REC of the measures taken and the circumstances giving rise to those measures.

### Frequency and plans for auditing trial conduct {23}

The trial shall permit direct access to participant’s records and source documents for the purposes of monitoring, auditing or inspection by the Sponsor, authorised representatives of the Sponsor, NHS, Regulatory Authorities and RECs.

### Plans for communicating important protocol amendments to relevant parties (e.g. trial participants, ethical committees) {25}

All protocol amendments will be approved by the funder, Clinical Trials Unit, EbE panel and ethics committee before implementation. Substantial amendments will also need to be approved by oversight committees. All protocol amendments will be communicated to associated PMHS who will also issue local approval.

### Dissemination plans {31a}

We will use a multi-modal dissemination plan to share the outcomes of the trial to ensure they reach a range of targeted audiences, including academics, front-line NHS staff and birthing parents with perinatal mental health difficulties. The study team will publish the findings in a series of peer-reviewed academic papers and aim to present the findings at relevant national and international conferences. The study findings and implications will also be shared in other mediums including blogs, infographics and social media content. The team will share the findings of the trial via newsletters, events and listservs connected to the NHS England national perinatal and CYP mental health transformation programme, the regional NHS Strategic Clinical Networks for Perinatal Mental Health, the BPS Faculty for Perinatal Psychology, the UK and Ireland Marce Society, the Parent-Infant Foundation Network, the Maternal Mental Health Alliance and the 1001 Critical Days All-Party Parliamentary Group. The study team also plan to hold an Expert by Experience event to share the findings with the study participants and the wider EbE community, which will be led by the study’s EbE panel.

## Discussion

To the best of our knowledge, the COSI study is the first fully powered randomised controlled trial of the clinical effectiveness and cost-effectiveness of COS-P for birthing parents with complex and severe mental health difficulties in the perinatal period and will include the largest process evaluation of its kind worldwide.

The treatment and prevention of parental psychopathology and parent-infant relationship difficulties in the perinatal period are areas of key concern to the NHS and public services globally, but there are numerous gaps in the evidence of effective psychological interventions during this critical time in family life. The proposed research aims to address the specific research recommendations in the NICE antenatal and postnatal guideline recommendations on evaluating group programmes in the perinatal period: interventions that target difficulties in both parental psychopathology and the parent-infant relationship difficulties; and transdiagnostic approaches of intervention. The study, if effective in treating parental psychopathology, will lead to improved short- and long-term outcomes for birthing parents and their children across a range of domains, including improved psychiatric, educational and physical health outcomes.

The study has been designed with dissemination in mind. Our choice of the CORE-OM as the primary outcome was motivated by it being one of the most widely used outcome measures in secondary care mental health services, including PMHS, and as such, is familiar to service managers, as well as local and national commissioners. It is also compatible with the national Mental Health Service Dataset. In this way, we hypothesise that any changes detected on it as a result of this trial will be highly compelling to key decision-making stakeholders and have the potential to positively impact clinical practice.

This is a study that has the voice of birthing parents at its foundation; experts by experience have been involved in the study from inception to grant writing to study delivery. The idea for the trial came from the recommendation of experts by experience. The study team benefit from a large, diverse, activated expert by experience panel who are a core part of the study team and we have three members of the EbE panel being trained as qualitative researchers. In this way, we ensure that all aspects of study delivery reflect on the experience of—and benefit to—birthing parents themselves.

## Trial status

The current protocol version is 5.0 dated 08/12/2022. Participant recruitment began on 04/01/2021 and is expected to be completed by 31/08/2023.

## Supplementary Information

**Additional file 1. **

**Additional file 2.** 

**Additional file 3.** 

**Additional file 4.** 

**Additional file 5.** 

**Additional file 6.** 

**Additional file 7.** 

## Data Availability

The anonymised quantitative datasets (e.g. questionnaire data but not video recordings) generated during the current study will be available as de-identified data upon request from Peter Fonagy and Camilla Rosan (peter.fonagy@annafreud.org; camilla.rosan@annafreud.org), beginning 12 months and ending 5 years after the primary publication and pre-planned secondary analysis following approval of a methodologically sound proposal and a signed data-sharing agreement. The transcripts from the interviews and focus groups will not be made available because, whilst the names of places and people will have been removed, the combination of contextual information given by participants could compromise their anonymity if the transcripts were available in their entirety.

## Abbreviations

AE: Adverse event
APPG: All-Party Parliamentary Group
ASQ-3: Ages and Stages Questionnaire-3
ASQ-SE: Ages and Stages Questionnaire-Social-Emotional
CORE-10: Clinical Outcomes in Routine Evaluation-10
CORE-OM: Clinical Outcomes in Routine Evaluation-Outcome Measure
COS-P: Circle of Security-Parenting
CI: Chief Investigator
CONSORT: Consolidating Standards of Reporting Trials
CRN: Clinical Research Network
CSRI: Client Service Receipt Inventory
DBL: Database Lock
DERS: Difficulties in Emotion Regulation Scale
DMEC: Data Monitoring and Ethics Committee
EbE: Expert by Experience Panel
EDC: Electronic Data Capture
EPDS: Edinburgh Postnatal Depression Scale
EQ-5D-5L: EuroQol-5D
GP: General Practitioner
HTA: Health Technology Assessment
ICC: Intra-Cluster Correlation
ICF: Informed consent form
IP: Intellectual property
MAR: Missing at Random
MCID: Minimal Clinically Important Difference
MNAR: Missing Not at Random
NICE: National Institute for Health and Care Excellence
NIHR: National Institute for Health and Care Research
PBQ: Postpartum Bonding Questionnaire
PIS: Participant Information Sheet
PMHD: Perinatal Mental Health Difficulty
PMHS: Perinatal Mental Health Service
QALY: Quality-adjusted life year
RCT: Randomised Controlled Trial
REC: Research Ethics Committee
SAE: Serious adverse event
