# Supplementary material for: The COSI trial: a study protocol for a multi-centre, randomised controlled trial to explore the clinical and cost-effectiveness of the Circle of Security-Parenting Intervention in community perinatal mental health services in England
Source: Trials. 2023 Mar 14;24:188. doi: 10.1186/s13063-023-07194-3 (PMC10012495; doi:10.1186/s13063-023-07194-3)
Supplement: Supplementary file 1 — Additional file 1. [file 13063_2023_7194_MOESM1_ESM.docx]

12 month follow-up

7 month follow-up

3 month follow-up

7 month follow-up

12 month follow-up

Experience survey and Interviews

3 month follow-up

Treatment as Usual

Circle of Security-Parenting

Inclusion

Randomisation

Screening
