# Supplementary material for: The COSI trial: a study protocol for a multi-centre, randomised controlled trial to explore the clinical and cost-effectiveness of the Circle of Security-Parenting Intervention in community perinatal mental health services in England
Source: Trials. 2023 Mar 14;24:188. doi: 10.1186/s13063-023-07194-3 (PMC10012495; doi:10.1186/s13063-023-07194-3)
Supplement: Supplementary file 2 — Additional file 2. [file 13063_2023_7194_MOESM2_ESM.docx]

**Appendix 3. COSI Trial Internal Pilot**

The internal pilot aims to review the trial data at an early stage in the study to ensure that the trial is able to deliver on its objectives within the planned timetable and budget. Completed at month 19, 12 months after recruitment has started, the internal pilot will evaluate 5 criteria across all of the Trial sites. In line with the recommendations in Avery et al (2017), a green/amber/red traffic light system will be used to advise progression from the internal pilot to the main trial.

*Criteria overseen by the Trial Steering Committee (TSC) with discussion with the funder:*

1. Recruitment rate overall and by trial site; and
2. Retention to the trial at 3 months (additionally follow-up at 7 & 12 months will be
    monitored throughout by DMEC)

| **TSC progression criteria** | Recruitment Criteria* | Retention Criteria |
| --- | --- | --- |
| ***RED:***  Do not progress to main trial | Average recruitment per recruitment cohort** is less than 3 per site (58%) | Retention at 3 months is less than 50% |
| ***AMBER:***  Explore methods to increase recruitment and/or retention | Average recruitment per recruitment cohort is between 3 (58%) and 5.2 (100%) per site | Retention at 3 months is between 50% and 95%*** |
| ***GREEN:***  progress to main trial | Average recruitment per recruitment cohort >= 5.2 per site (100%) | Retention at 3 months is greater than or equal to 95%* |

*all sites will be held to the same criteria

** This trial does not have monthly recruitment. Recruitment will take place in eight periodic 4-week recruitment cohorts.

***5% missing at 3 months is incorporated

*Criteria overseen for the intervention arm by the Data Monitoring and Ethics Committee (DMEC; who will be unblinded to data by arm):*

- 1. Average time to first intervention session from randomisation;
  2. Adherence to the planned intervention sessions by participants; and
  3. Fidelity of delivery of the intervention by healthcare professionals.

 The DMEC’s assessment will be based on a credible intervention dose being received, contamination issues and monitoring in prescribing of ‘treatment as usual’ therapies in the control arm that would be cause for concern. Their recommendation will be at their discretion and with evaluation across the multiple outcomes, but they will however be provided the above framework to assist their decision-making.

| **DMEC subjective review criteria** | **Time to first intervention** | **Adherence to the planned intervention** | **Fidelity of delivery** |
| --- | --- | --- | --- |
| ***RED:***  Recommend not to progress to main trial | Average time >8 weeks | Average dosage < 4 sessions | Fidelity < 50% |
| ***AMBER:***  Recommend changes to operational aspects of the main trial | Average time is between 4 and 8 weeks | Average dosage is between 4 and 6 sessions | Fidelity is assessed as between 50-75% |
| ***GREEN:***  progress to main trial with no changes | Average time is < 4 weeks | Average dosage > 6/10 sessions | Fidelity is assessed as > 75% |

If the trial progresses beyond the pilot the DMEC will continue to monitor these outcomes throughout the trial.

*Outcomes of the Internal Pilot*
The following outcomes will be evaluated:

- **Recruitment rates** (per recruitment cohort per site and overall)
- **Retention rates** (to the trial overall at the 3-month follow-up at each site)
- **Time to first COS-P session in the intervention arm** (average time between randomisation of participant and delivery of first COS-P session in intervention group)
- **Adherence to the planned intervention** (assessed via an independent review of video recordings of each facilitator’s third COS-P group)
- **Fidelity of Delivery** (as detailed in section ‘Strategies to improve adherence to interventions {11c}’ of the paper)

**References**

Avery KN, Williamson PR, Gamble C, et al., Informing efficient randomised controlled trials: exploration of challenges in developing progression criteria for internal pilot studies, BMJ Open 2017: 7(2).
