# Supplementary material for: The COSI trial: a study protocol for a multi-centre, randomised controlled trial to explore the clinical and cost-effectiveness of the Circle of Security-Parenting Intervention in community perinatal mental health services in England
Source: Trials. 2023 Mar 14;24:188. doi: 10.1186/s13063-023-07194-3 (PMC10012495; doi:10.1186/s13063-023-07194-3)
Supplement: Supplementary file 3 — Additional file 3. [file 13063_2023_7194_MOESM3_ESM.docx]

**Appendix 4. COSI Trial Screening Measures**

The following outcome measures will be used to confirm eligibility for the trial.

1. **Postpartum Bonding Questionnaire (PBQ)**

   The PBQ (Brockington, Fraser & Wilson, 2006) is a self-administered measure designed to provide an early indication of disorders within birthing parent-infant relationships through the assessment of a birthing parent’s feelings and attitudes towards her infant. The PBQ includes 25 items which are each presented as a statement, e.g. “I feel happy when my baby smiles or laughs”. Respondents are asked to indicate how often each statement applies to them on a 6-point scale ranging from “Always” to “Never”. Scores on the PBQ are provided as a total score of bonding difficulties, and on four subscales: Impaired Bonding, Rejection and Pathological Anger, Infant-Focused Anxiety, and Incipient Abuse. In particular, the Impaired Bonding subscale is the PBQ’s General Factor used to identify a general problem within the birthing parent-infant relationship. This measure is frequently used in research on birthing parent-infant bonding with postpartum populations (Brockington et al., 2001; Reck et al., 2006; Edhborg, Nasreen & Kabir, 2011), has been validated for use in several languages and cultures (Garcia-Esteve et al., 2016; Siu et al, 2010), and has demonstrated acceptable reliability and reasonable validity (Overall α = .76; Subscale α range .63 -.79) (Wittowski, Wieck & Mann, 2007).

1. **Clinical Outcomes in Routine Evaluation- 10 (CORE-10)**

The CORE-10 **(Barkham et al., 2013)** is a session-by-session monitoring tool exploring respondents recent mental health difficulties. Developed as a shorter version of the Clinical Outcomes in Routine Evaluation – Outcome Measure (CORE-OM; Barkham et al., 2013), the CORE-10 includes 10-items presented as statements (e.g., “I have felt tense, anxious or nervous”). Respondents are asked to indicate the frequency that these statements have applied to them over the last week on a 5-point scale ranging from “Not at All” to “Most or All of the Time”. Scores on the CORE-10 estimate psychological distress as an indication of mental health, and these scores are broadly interpreted as ranging from healthy (total score of 0-5) to severe psychological distress (score of 25-40). The CORE-10 has been shown to have good psychometric properties with good internal reliability (α = .90) (Barkham et al., 2013) and has been validated for use during pregnancy (Coates et al., 2020). The CORE-10 is routinely used with people presenting common mental health properties in primary care settings.

**References**

Barkham M, Bewick B, Mullin T, Gilbody S, Connell J, Cahill J, Mellor-Clark J, Richards D, Unsworth G, Evans C. The CORE‐10: A short measure of psychological distress for routine use in the psychological therapies. Counselling and Psychotherapy Research. 2013 Mar;13(1):3-13.

Brockington IF, Fraser C, Wilson D. The Postpartum Bonding Questionnaire: a validation. Arch Womens Ment Health. 2006;9(5):233-42.

Brockington IF, Oates J, George S, Turner D, Vostanis P, Sullivan M, Loh C, Murdoch C. A screening questionnaire for mother-infant bonding disorders. Archives of women's mental health. 2001 Mar;3:133-40.

Coates R, Ayers S, de Visser R, Thornton A. Evaluation of the CORE-10 to assess psychological distress in pregnancy. Journal of Reproductive and Infant Psychology. 2020 May 26;38(3):311-23.

Edhborg M, Nasreen HE, Kabir ZN. Impact of postpartum depressive and anxiety symptoms on mothers’ emotional tie to their infants 2–3 months postpartum: a population-based study from rural Bangladesh. Archives of women's mental health. 2011 Aug;14:307-16.

Evans C, Connell J, Barkham M, et al., Towards a standardised brief outcome measure: psychometric properties and utility of the CORE–OM, Brit J Psychiatry 2002: 180(1); 51-60.

Garcia-Esteve L, Torres A, Lasheras G, Palacios-Hernández B, Farré-Sender B, Subirà S, Valdés M, Brockington IF. Assessment of psychometric properties of the Postpartum Bonding Questionnaire (PBQ) in Spanish mothers. Archives of women's mental health. 2016 Apr;19:385-94.

Reck C, Klier CM, Pabst K, Stehle E, Steffenelli U, Struben K, Backenstrass M. The German version of the Postpartum Bonding Instrument: psychometric properties and association with postpartum depression. Archives of women's mental health. 2006 Sep;9:265-71.

Siu BW, Ip P, Chow HM, Kwok SS, Li OL, Koo ML, Cheung EF, Yeung TM, Hung SF. Impairment of mother-infant relationship: validation of the Chinese version of postpartum bonding questionnaire. The Journal of nervous and mental disease. 2010 Mar 1;198(3):174-9.

Wittkowski A, Wieck A, Mann S. An evaluation of two bonding questionnaires: a comparison of the Mother-to-Infant Bonding Scale with the Postpartum Bonding Questionnaire in a sample of primiparous mothers. Archives of women's mental health. 2007 Aug;10:171-5.
