# Supplementary material for: The COSI trial: a study protocol for a multi-centre, randomised controlled trial to explore the clinical and cost-effectiveness of the Circle of Security-Parenting Intervention in community perinatal mental health services in England
Source: Trials. 2023 Mar 14;24:188. doi: 10.1186/s13063-023-07194-3 (PMC10012495; doi:10.1186/s13063-023-07194-3)
Supplement: Supplementary file 4 — Additional file 4. [file 13063_2023_7194_MOESM4_ESM.docx]

**Appendix 5. COSI Trial Outcome Measures**

The following secondary outcome measures are used within the trial.

1. **Demographics** 
   A 25-item measure used to report contextual factors within the study sample was developed by the study team for this project. Items are considered under three sections: You and Your Youngest Child, Pregnancy and Mode of Birth, and Service Receipt. Items included in the scale report on participant sexual orientation, religion, disability, socioeconomic information, complications during pregnancy and birth, previous pregnancies, and maternal history of mental health difficulties.
2. **Postpartum Bonding Questionnaire (PBQ) (Brockington, Fraser & Wilson, 2006)** 
   The PBQ is a 25-itemmeasure designed to provide an early indication of disorders within birthing parent-infant relationships through the assessment of a mother’s feelings and attitudes towards her infant. Items within the PBQ are presented as statements (e.g., “I feel close to my baby”) with 6 response options ranging from “Always” to “Never”. Responses on the PBQ are scored to provide an overall score of bonding difficulties, and a score on four subscales: Impaired Bonding, Rejection and Pathological Anger, Infant-Focused Anxiety, and Incipient Abuse. The PBQ is frequently used in research on mother-infant bonding within postpartum populations, and is reported to have high validity and reliability (α = .76) (Brockington et al, 2001; Reck et al., 2006; Edhborg et al., 2011; Wittkowski et al., 2007).
3. **The Difficulties in Emotion Regulation Scale (DERS) (Gratz & Roemer, 2004)** 
   The DERS is a self-report measure of perceived maternal emotion regulation ability. The scale comprises 36 statements of emotion regulation such as “when I'm upset, I acknowledge my emotions”. Respondents are asked to report the frequency of which these statements apply to them on a scale ranging from 1 (Almost Never) to 5 (Almost Always). Scoring of the DERS provides a total score of emotion regulation with higher scores indicating greater difficulties in regulation. Six subscales are also included within the measure: Nonacceptance of Emotional Responses, Difficulty Engaging in Goal-Directed Behaviour, Impulse Control Difficulties, Lack of Emotional Awareness, Limited Access to Emotion Regulation Strategies, and Lack of Emotional Clarity. Validation of the DERS reported high validity and internal consistency both overall (α = .93), and within each subscale (α = .80) (Gratz & Roemer, 2004).
4. **Ages and Stages Questionnaire-3 & Socio-emotional (ASQ-3 & SE) (Squires, Bricker & Potter, 2009; Squires, Bricker & Twombly, 2015)**
   The Ages and Stages Questionnaires are tools for assessing infant development. The ASQ-3 is used specifically to assess and screen global infant development in communication, motor and cognitive areas. There are 20 versions of the ASQ-3 available to cover an age range between 1 and 66 months of age. An example of an item in the ASQ-3 is “does your baby chuckle softly?”, however, the items and number of items within each version of the scale differ in order to ensure that they are appropriate for infants within the specified age range. The ASQ-3 is commonly used across many cultures and has demonstrated high test-retest reliability and predictive validity in identifying infants at risk of developmental delay (Rothstein, Miskovic & Nitsch, 2017).

   The ASQ-SE has an extremely similar format to the ASQ-3 but instead focuses specifically on infant’s social and emotional development. The ASQ-SE reports on infant’s development from 2 – 65 months and there are 8 variations of the scale available to cover these age ranges. An example item from one of these questionnaires is “does your baby smile at you and other family members?”. The ASQ-SE is commonly used as a measure of social and emotional development in infants, however, has weak reliability and validity (de Wolff, Theunissen, Vogels & Reijneveld, 2013).
5. **NICHD Sensitivity Scales (Owen, 1992)**  
   Participants complete a guided parent-infant interaction during each study data collection visit, during which they are asked to play with their child without any toys, to read a book with their child, and to change a piece of their child’s clothing. These interactions are audio and video recorded and coded by two blinded, independent raters. The NICHD Sensitivity Scales are used as the coding scheme for these recordings. The NICHD Sensitivity Scales are a measure of maternal sensitivity designed for use in parents with infants aged up to 24 months old and measure sensitivity through behaviours relating to the proportion of distress signals responded to, latency of the response, and the appropriateness of the response.
6. **Strange Situation Procedure (SSP) (Ainsworth, Blehar & Waters, 1978)**
   The SSP is the gold standard assessment of attachment security in infancy and has been validated for use across many cultures (van IJzendoorn & Kroonenberg, 1988). The SSP is audio and video recorded, and these recordings are coded to classify infant’s attachment security into one of the 9 attachment subgroups provided: Insecure Avoidant Subgroups A1 and A2, Secure Reserved Subgroup B1, Secure Inhibited Subgroup B2, Secure Balanced Subgroup B3, Secure Reactive Subgroup B4, Insecure Resistant Subgroup C1, Insecure Resistant Subgroup C2, and Insecure Disorganised Group D. The SSP is completed at the 12-month follow-up visit of the study and all participants are asked to wear a heart rate monitor during the task. State Space Grids coding grids are also used during the task to evaluate co-regulation of emotion.
7. **EuroQol- 5 Dimension (EQ-5D-5L) (EuroQol Group, 1990)**
   The EQ-5D-5L is a 6-item preference-based health-related quality of life measure to compute quality-adjusted life-years (QALYs) for economic evaluation.  The first 5 items in the scale require the respondent to rate their health today across 5 dimensions (mobility, self-care, usual activities pain or discomfort, anxiety and depression) on a 5-point scale. The final question in the EQ-5D-5L requires the participant to rate their overall health today on a scale from 0 (worst health imaginable) to 100 (best health imaginable).
8. **Client Service Receipt Inventory (CSRI) (Beecham & Knapp, 2001)** 
   The CSRI is a 24-item questionnaire designed to evaluate participant’s service use. The CSRI collects some sociodemographic information (gender identity, relationship status, ethnicity, employment and income, education, and living situation) and enquires into participant’s health service usage, including use of health and social care services and current medications.
9. **Treatment as Usual (TAU)**
   In order to best understand the usual treatment received by all participants in the study and to map the variations in usual treatment offered by each trial site, an additional item focusing on TAU was created by the research team. This question regarding TAU is included in the CSRI and asks participants to report any mental health or psychological support received since their previous study data collection visit. Participants are asked to report if they have received any of the following: review of mental health medication, assessment or review of mental health, informal check-in about mental health, and psychological and/or support intervention for mental health. Additional information is then collected for any type of support reported (e.g., number of sessions, contact time, professional seen, and format of sessions).
10. **Childhood Trauma Questionnaire-Short Form (CTQ-SF) (Bernstein et al., 2003)**
    The CTQ-SF is a 28-item version of the Childhood Trauma Questionnaire (Bernstein et al., 1998), and is used to screen participant’s history of maltreatment. Each item is presented as a statement (e.g., “I didn’t have enough to eat”) with 5 response options ranging from “Never True” to “Very Often True”. The CTQ-SF has demonstrated good validity and has been validated for use within clinical populations (Bernstein et al., 2003; Kongerslev et al., 2019) . Childhood trauma and maltreatment reported in the questionnaire is considered within 5 subscales: emotional abuse, physical abuse, sexual abuse, emotional neglect, and physical neglect.
11. **Conflict and Problem-Solving Strategy Scales – Short Form (CPS-SF) (Helland et al., 2021)**
    The CPS-SF is an 18-item measure of inter-parental conflict behaviours developed from the original, 88-item, Conflict and Problem-Solving Strategy Scales (Kerig, 1996). Items on the CPS-SF reflect different possible conflict resolution strategies such as “listen to the other’s point of view” or “make accusations”. For each of these items, respondents report the frequency with which they and their partner each use these strategies on a scale from 0 (Never) to 3 (Often). The CPS-SF also includes 6 subscales of conflict behaviours on which responses are scored: Cooperation, Avoidance, Child Involvement, Stalemating, Verbal Aggression, and Physical Aggression. The scale has been validated for use within clinical and various family structures (Helland et al., 2021), and was felt to be acceptable by the COSI Study’s EbE panel.
12. **Adverse Events Questionnaire**  
    Adverse Events and Serious Adverse Events are monitored throughout the course of the study. A 2-item questionnaire is completed during the data collection visits to allow participants to report any adverse events experienced during the study. This questionnaire includes a list of possible physical (e.g., eye strain) and social adverse events (e.g., involvement of social care with the family) and allows a free-text response for any additional events to be reported.
13. **Participation Declination Survey**
    To understand the factors and barriers influencing a decision not to take part in the trial and to ensure that participation is as accessible as possible, parents who decline to take part in the trial will be asked to complete a short survey about their decision.

**References**

Ainsworth MD, Blehar MC, Waters E, Wall S. Strange situation procedure. Clinical Child Psychology and Psychiatry. 1978.

Beecham J, Knapp M. Costing psychiatric interventions. In: Thornicroft G, editor. Measuring Mental Health Needs. 3rd ed. London: Gaskell/Royal College of Psychiiatrists; 2001; 200-24.

Bernstein DP, Fink L, Handelsman L, et al., Childhood trauma questionnaire. Assessment of family violence: a handbook for researchers and practitioners, 1998.

Bernstein DP, Stein JA, Newcomb MD, et al., Development and validation of a brief screening version of the Childhood Trauma Questionnaire, Child Abuse & Neglect 2003: 27(2); 169-90.

Brockington IF, Fraser C, Wilson D. The Postpartum Bonding Questionnaire: a validation. Arch Womens Ment Health. 2006;9(5):233-42.

Brockington IF, Oates J, George S, Turner D, Vostanis P, Sullivan M, Loh C, Murdoch C. A screening questionnaire for mother-infant bonding disorders. Archives of women's mental health. 2001 Mar;3:133-40.

Gratz KL, Roemer L., Multidimensional assessment of emotion regulation and dysregulation: development, factor structure, and initial validation of the difficulties in emotion regulation scale, J Psychopathol Behav Assess 2004: 26(1); 41-54.

Helland MS, Holt T, Gustavson K, Larsen L, & RØysamb E, Validation and short-form development of Conflict and Problem-solving Strategy Scales, J Fam Studies 2021.

Kerig PK, Assessing the links between interparental conflicts and child adjustment: the Conflict and Problem-Solving Scales, J Fam Psych 1996: 10(4); 454-473.

Kongerslev MT, Bach B, Rossi G, Trauelsen AM, Ladegaard N, Løkkegaard SS, Bo S. Psychometric validation of the Childhood Trauma Questionnaire-Short Form (CTQ-SF) in a Danish clinical sample. Child abuse & neglect. 2019 Aug 1;94:104026.

Owen MT. The NICHD study of early child care mother–infant interaction scales. Unpublished manuscript, Timberlawn Psychiatric Research Foundation, Dallas, TX. 1992.

Reck C, Klier CM, Pabst K, Stehle E, Steffenelli U, Struben K, Backenstrass M. The German version of the Postpartum Bonding Instrument: psychometric properties and association with postpartum depression. Archives of women's mental health. 2006 Sep;9:265-71.

Rothstein A, Miskovic A, Nitsch K. Brief review of psychometric properties and clinical utility of the Ages and Stages Questionnaires, for evaluating pediatric development. Archives of Physical Medicine and Rehabilitation. 2017 Apr 1;98(4):809-10.

Squires J, Bricker D, Potter L. Ages & Stages Questionnaires®, Third Edition (ASQ®-3): A Parent-Completed Child Monitoring System. Baltimore: Paul H. Brookes Publishing Co., Inc.; 2009

Squires J, Bricker D, Twombly E. Ages & Stages Questionnaires®: Social-Emotional, Second Edition (ASQ®:SE-2): A Parent-Completed Child Monitoring System for Social-Emotional Behaviors. Baltimore: Paul H. Brookes Publishing Co., Inc.; 2015.

The EuroQol Group, EuroQol - a new facility for the measurement of health-related quality of life. Hlth Policy 1990: 16(3); 199-208.

Van IJzendoorn MH, Kroonenberg PM. Cross-cultural patterns of attachment: A meta-analysis of the strange situation. Child development. 1988 Feb 1:147-56.

de Wolff MS, Theunissen MH, Vogels AG, Reijneveld SA. Three questionnaires to detect psychosocial problems in toddlers: A comparison of the BITSEA, ASQ: SE, and KIPPPI. Academic pediatrics. 2013 Nov 1;13(6):587-92.

Wittkowski A, Wieck A, Mann S. An evaluation of two bonding questionnaires: a comparison of the Mother-to-Infant Bonding Scale with the Postpartum Bonding Questionnaire in a sample of primiparous mothers. Archives of women's mental health. 2007 Aug;10:171-5.
