# Supplementary material for: The COSI trial: a study protocol for a multi-centre, randomised controlled trial to explore the clinical and cost-effectiveness of the Circle of Security-Parenting Intervention in community perinatal mental health services in England
Source: Trials. 2023 Mar 14;24:188. doi: 10.1186/s13063-023-07194-3 (PMC10012495; doi:10.1186/s13063-023-07194-3)
Supplement: Supplementary file 5 — Additional file 5. [file 13063_2023_7194_MOESM5_ESM.docx]

**The COSI Study**

**survey for use with parents (v3 30/06/2022)**

Note: the survey is viewable here for completion in Qualtrics: Qualtrics Survey | Qualtrics Experience Management

1. Thank you for taking part in this survey about your experiences of the Circle of Security parenting programme. Your experience and reflections of the sessions are a valuable part of understanding and improving perinatal mental health services in the future, even if you did not attend all the sessions. There are no right or wrong answers. Your responses to this survey, personal information and identity will be kept confidential. Your responses will not impact your relationship with any services. The survey will focus on the group sessions you have taken part in (both online and in-person), but you will have also received other types of support from the perinatal mental health service so if that is relevant then please also tell us about that in the box at the end of the survey.

a. I confirm that I understand and would like to continue with the survey and complete it myself [🡪 directs to main questions]

b. I confirm that I understand and would like to do the survey with a member of the research team by phone [🡪directs to thank you screen]

c. I do not want to continue with the survey [🡪directs to thank you screen]

Instructions for Survey:

2. The survey will take no more than approximately 20 minutes to complete and can be completed at a time that is suitable to you. If you wish to take a break, you are able to exit the survey and return to it at a later time in that week but please complete it using the same device (e.g. mobile or tablet). If there are certain questions that you do not want to answer, you are able to skip these questions.

**Your experience of the Circle of Security Parenting Programme**

3. Each session lasted about 90 minutes. Did you find the length of each session:

1. too long
2. about right
3. too short

4. Any comments

5. Did you find the size of your group:

1. too many
2. about right
3. too small

6. Any comments

7. If there was an option to access the group online or face-to-face (in-person), which would you prefer?

1. online
2. face-to-face (in-person)
3. no preference
4. a combination of online and face-to-face

8. Any comments

9. Would you prefer having the option for babies to be present at the group?

1. I would prefer not to have babies attend sessions
2. I would prefer to have babies attend all the sessions
3. I would prefer to have babies attend some of the session (e.g. part of the sessions of particular weeks)
4. not sure/ don't know

10. Any comments (including about older children)

11. Would you prefer having the option for a person supporting you in parenting (e.g. partner, parent, close friends) to attend the group?

1. yes, I would prefer that they can attend
2. no, I would prefer that they cannot attend
3. not sure / don't know

12. Any comments

13. Please tell us anything that made it easier for you to attend

-----------------------------------------------------------------------------

14. Please tell us anything that made it harder for you to attend

-----------------------------------------------------------------------------

15. How helpful did you find it to be in a group with other parents?

1. very helpful
2. helpful
3. neither helpful or unhelpful
4. unhelpful
5. very unhelpful

16. Any Comments

17. How helpful did you find the group facilitator(s)?

1. very helpful
2. helpful
3. neither helpful or unhelpful
4. unhelpful
5. very unhelpful

18. Any Comments

19. Considering your baby’s age, how helpful did you find the material and concepts covered?

1. very helpful
2. helpful
3. neither helpful or unhelpful
4. unhelpful
5. very unhelpful

20. Any Comments

**Overall**

21. Overall, how satisfied were you with the Circle of Security Parenting Programme?

1. very satisfied
2. satisfied
3. neither satisfied or unsatisfied
4. unsatisfied
5. very unsatisfied

22. Any Comments

23. What did you find most useful about the Circle of Security parenting programme? (List up to three)

-----------------------------------------------------------------------------

24. What did you find least useful about the Circle of Security parenting programme? (List up to three)

-----------------------------------------------------------------------------

**Further Comments**

25. Is there anything else you would like to add about your experiences of the Circle of Security parenting programme?

-----------------------------------------------------------------------------

26. Was there any other support from the perinatal mental health team outside of the Circle of Security parenting programme that affected your experience of the programme? (either positively or negatively)

-----------------------------------------------------------------------------

**Sharing your views and experiences in more detail**

27. Are you interested in taking part in a one-to-one interview about your views and experiences of the Circle of Security parenting programme? If so, you will be contacted by someone from the research team (at the Anna Freud Centre or the University of Huddersfield) who is doing the interviews. They will be able to answer any questions you may have before deciding whether you would like to take part. Anyone who is in interviewed will be offered a £20 voucher to thank them for their time. Unfortunately it is not possible to interview everyone. To help describe whose views have been shared in the study, we link the interviews with information from the main study, including background information that you have shared in the study, your survey responses and information about the group that you attended (e.g. the number of sessions attended, the study site, whether the group used an interpreter). This is done by using your anonymous study code number. No identifying information (e.g. name) will be used in reporting the study’s findings.

1. Yes
2. No
