# Supplementary material for: The COSI trial: a study protocol for a multi-centre, randomised controlled trial to explore the clinical and cost-effectiveness of the Circle of Security-Parenting Intervention in community perinatal mental health services in England
Source: Trials. 2023 Mar 14;24:188. doi: 10.1186/s13063-023-07194-3 (PMC10012495; doi:10.1186/s13063-023-07194-3)
Supplement: Supplementary file 6 — Additional file 6. [file 13063_2023_7194_MOESM6_ESM.docx]

**The COSI Study**

**Process evaluation topic guide – semi-structured interview schedule for use with parents (v2.1 04/04/2022)**

| Once informed consent has been secured, the following broad topics will be discussed. It is common in qualitative work to iteratively develop topics and questions as new ideas emerge from early data collection. We may therefore add new topics as the interviews progress and data collection continues, but the emphasis will remain on parents’ views and experiences of the intervention. The exact order and phrasing of questions will remain flexible, as is usual with semi-structured interviews. The interviewer may also draw on the participant’s comments from the survey, using these as prompts for further discussion; not all questions will necessarily be asked of all participants. The interview will be conducted with participants who vary in the number of sessions attended and the questions will be used flexibly in recognition of this. |
| --- |

**Your experience and reflections of the Circle of Security parenting programme isa valuable part of understanding and improving perinatal mental health services. There are no right or wrong answers. Your responses to this interview, personal information and identity are confidential. You will not be identified in any report and your responses will not impact your relationship with any services you are in contact with.**

**I will begin with some questions about the format of the programme, and also ask you about any changes that you have noticed in yourself and your relationships. The focus of the interview is on your views and experiences of the Circle of Security parenting programme but there will also be the chance to talk about any other support you have had from the service, if you feel this is relevant. If there are any questions that you would prefer not to answer, we can skip those questions.**

**Introduction**

1. How would you describe the Circle of Security Programme to another parent being offered this programme in a perinatal mental health service?

***Barriers/facilitators***

2. I’d like to ask you about anything that made it easier/harder for you to get to the sessions.

a. (If applicable) In the survey, you mentioned xxxxx. Can you tell me more about this? [*Prompts: explore practical aspects (eg, childcare, internet connection/call quality, travel) and emotional/ psychological aspects, current symptoms).* How did this vary for in-person compared with online sessions?

b. (If applicable) You attended xx sessions of the 10 sessions. With the sessions that were missed, was there anything in particular that got in the way? Do you have any suggestions for what would have helped?

3. I’d like to ask you what it was like to do this (mostly) online.

a. How did this compare with your expectations?

b. Did you do this from your own home?

c. What was the best/hardest aspect of being online? Do you have any suggestions for how this could be improved? [*link with survey where relevant, e.g. you mention x was a challenge, can you tell me more]*

4. I’d like to hear about what it was like to do this as a group programme with other parents.

a. How did you find taking part in group discussions? How did this compare with your expectations?

b. (If applicable) what was it like to have the group include an interpreter? / to be supported by an interpreter? *[If individually using an interpreter then this will be considered throughout the interview]*

c. Would you have wanted other opportunities to meet with the other parents who were on the programme?

d. If applicable (dependent on survey response), explore views on the group size.

e. Overall, do you think having this as a group programme has been more/less helpful than if it were offered on an individual basis, and why?

5. Can you tell me about your relationship with your facilitator?

a. What was helpful/unhelpful? In what ways?

b. To what extent did you feel able to talk freely? *[Prompts: Explore feelings of safety and exploration. Explore any online vs. in-person comparisons (but mindful the content of these sessions may have varied).]*

6. Can you tell me about anything else that made it easier/harder to *follow* the sessions (e.g following the ideas, concentrating)? *[Prompts: explore emotional/psychological aspects (e.g. distress/discomfort/concentration, trauma/memories and flashbacks, shame, baby being present or absent); explore feelings of safety – with facilitator and with other parents/group dynamics]*

7. To what extent do you feel the materials (videos and worksheets) and concepts fitted with your ‘family culture and values’? *[Prompt: explore examples, e.g. who’s in a family, any cultural differences, difference in our communities; note: we will discuss suitable prompts further with the EbE panel, Nic & Ruth]*

8. [linking to the survey] You said x about how relevant you found the materials and concepts in relation to your baby’s age. Please can you tell me more. *[Prompt: were there any particular elements that felt less/more useful with a young baby? Prompts: explore views and experiences of the workbook]*

9. Please tell me your views on the timing of this programme, for you and your individual circumstances. For example, was it too soon / not soon enough? In what way? *[Prompt: may include age of child(ren), level of symptoms, other work being completed first]*

10. Have you shared your experience of the Circle of Security Parenting Programme with anyone outside of the group? What did you tell them? (e.g. materials and concepts/ideas)? Who did you tell? (e.g. any ‘parenting partners’ (someone who supports you with parenting) - your partner/baby’s other parent, or other family members or friends)? Have you discussed with anyone outside of your family and friends (e.g. perinatal peer support workers, online parenting forums)? *[Prompts: can you tell me a bit about that? Were there any tensions? Have you shared any worksheets with others?]*

***Any changes (positive or negative) and what may have led to these (change mechanisms)***

I will now ask you about any changes (positive or negative) that you have noticed in different areas of your life and the extent to which you see these as linked to the Circle of Security parenting programme. As discussed in the beginning of this interview, there are no right or wrong answers.

11. (If applicable) What was the format of the ‘pre group session’ with the facilitator (eg 1:1/group)? What did you find helpful/unhelpful? In what way? What did you discuss with your facilitator in the pre-group conversation?

12. Thinking back to before the programme started, what were you hoping to get out of it? [If the reasons that are given relate to the trial (e.g. helping other women/services), explore if anything specific to them and personal benefit for them or their relationship with their baby.] Note: this question may then naturally invite consideration of whether these hopes were realised, exploring the next few sections and inviting any others.

13. Please can you tell me about any differences that you have noticed with your mood/mental health, whether positive or negative? What do you think may have led to these?

14. Please can you tell me about any changes (positive or negative) that you have noticed in how you feel about your parenting (as a parent/mother)? *[Prompts: what has this looked/felt like? Has your confidence as a parent changed? What do you think may have led to this?]*

15. Please can you tell me about any changes that you have made/noticed in your relationship or bond with your baby? *[Prompts: what has this looked like? What has made this easier/harder to do? Have you found this helpful? How do you think your child may have experienced this? Were there any changes that you would have liked to make but have felt unable to? ]*

16. Have you noticed any changes in how you understand your baby’s way of communicating their needs? *[Prompts using COS terminology: have you noticed any changes in how they “travel around the circle of security”, e.g. travelling away from you as their safe base, returning to you as their safe haven? what has this looked like? What do you think has influenced this? If the Circle of Security, what messages have stayed with you?]*

17. [linking to the survey] You said x about your views on having babies at the session. Please could you tell me a bit more about this. *[Prompts: do you think it is more or less helpful to have the babies present? Why (not)? Would you have any concerns about children being present? How do you think this may affect discussions? Explore with reference to baby’s age]*

18. Have you involved others in any changes that you’ve made in your relationship with your baby (i.e. ‘how you are’ with your youngest child); for example your partner or other family members or friends?) Can you give an example? *[Prompts: what has worked well? What has been difficult?]*

a. Would you have liked to have anyone attend the group sessions with you? If so, who (e.g. your partner/baby’s other parent, your own parent, or other family members or friends)? *[Prompts: do you think this is helpful? Why (not)? Would you have any concerns about others taking part? How do you think this may affect discussions?]*

19. (If applicable) Have you noticed any changes in your relationship with your other child(ren)? *[Prompts: what has this looked like? What has made this easier/harder to do? Have you found this helpful? How do you think your child(ren) may have experienced this?]*

a. (If applicable) Would you have found the Circle of Security parenting programme beneficial with your other children when they were this age? In what way? *[Prompts: explore if had mental health difficulties at that time and if had support]*

20. Have you noticed any changes in your relationships with other adults, for example your partner or anyone who supports you in parenting (‘parenting partners’)? *[Prompts: Could you please tell me more, what has this looked like? What has made this easier/harder to do? Have you found this helpful? How do you think they may have experienced this?]*

21. Have you received any other support or interventions from the perinatal mental team outside of Circle of Security which you feel has had an impact on any of the things that we have discussed?

***Overall***

22. [If unclear from survey and not yet covered] In the survey, you said you found xxxxxxxxxx most helpful. Please can you tell me more about this?

23. [If unclear from survey and not yet covered] In the survey, you said you found xxxxxxxxxx least helpful. Please can you tell me more about this?

24. Can you tell me about anything else that you think should be considered before making this programme available to more parents that are accessing specialist perinatal mental health services?

25. Do you have anything else that you would like to tell me about your experiences of the programme that we have not already covered?

Thank for time and continued contribution to the study.
