# Supplementary material for: The COSI trial: a study protocol for a multi-centre, randomised controlled trial to explore the clinical and cost-effectiveness of the Circle of Security-Parenting Intervention in community perinatal mental health services in England
Source: Trials. 2023 Mar 14;24:188. doi: 10.1186/s13063-023-07194-3 (PMC10012495; doi:10.1186/s13063-023-07194-3)
Supplement: Supplementary file 7 — Additional file 7. [file 13063_2023_7194_MOESM7_ESM.docx]

**The COSI Study**

**Process evaluation topic guide – focus groups with professionals**

1. Welcome and ground rules; reminder about withdrawal
2. Introductions (providing context and assisting transcription via longer ‘soundbite’): first name, role (intervener/supervisor), professional background, number of groups involved with during the study, whether have previous experience (pre-COS-P) of i) parent-infant work and ii) group work
3. What do you see as being the top 3 ‘ingredients’ to this intervention? Why? How do these ‘work’? *[may revisit at the end, following other discussions and seeking consensus]*
4. What do you see as the most important aspects of the facilitator role for the COS-P programme?
5. What have the main barriers and facilitators for you in providing this programme?
   1. Practical aspects, e.g.
      1. systems (e.g. IT, time (of day) etc, tablet library)
      2. expectations for parents (e.g. childcare)
      3. pacing of programme (10 weeks)
      4. ethos of service (e.g. whether parenting is seen as ‘core business’)
   2. How well do you feel the COS-P training prepared you for this role? *[Prompts: explore gaps, including particular dynamics, parent mental health difficulties etc]*
   3. What support have you needed alongside the role, so that you can be the ‘hands’? / offer a ‘holding environment’? Has anything affected your ability to do this? *[Prompts: explore gaps – but mindful of group composition; explore use of coaching; explore vulnerability amongst staff; ability to be safe haven / secure base; self-regulation needed for co-regulation / managing own shark music]*
   4. COS-P is a reflective parenting programme that requires facilitators to reflect on their own ‘State of Mind’ and ‘Shark Music’. What came up for you and how did you manage this? *[Prompts: explore use of fidelity journal]*
   5. How equipped have supervisors felt for this programme? *[Prompts: explore if programme-specific/any distinct elements, including newness with parent-infant work]*
   6. What professional background experience do you think is helpful in coming to facilitate this programme? *[e.g. may draw upon previous group work, previous parenting work; may also explore experience that is helpful for supervisors]*
   7. Do you think any personal background experience is helpful/unhelpful in coming to facilitate this?
6. Thinking about the range of parents that access specialist perinatal mental health services, their diverse circumstances and needs, who do you think the programme works best for? Why? What timing (e.g. infants aged 0-3, 3-6, 6-12 months)? *[explore similarities and differences in the group]*
7. Are there any needs/circumstances where you think the programme isn’t suitable? *[Prompts: diagnosis/presenting difficulties, symptom severity/being ‘stable enough’ concerning mental health, severe self-regulatory difficulties, age of child(ren), having multiple children to attend to, specific communication needs, cultural relevance, support from partner]*
   1. Would this be different if delivered individually instead of in a group format?
   2. Would this be different if delivered in-person?
8. Do you have any feedback about the workbook that is given to parents? *[Prompts: explore most/least helpful aspects; relevance for perinatal period/different ages; issues of accessibility]*
9. Do you think that the programme has any unhelpful/troublesome elements? Anything that doesn’t sit as comfortably with you? *[Prompts: consider any issues of access, of accessibility, of diversity/inclusivity (eg language/communication needs; neurodiversity), of trauma, exclusive dyadic focus is western construct]* Is any of this more/less challenging because of being online?
10. What do you think are the main barriers and facilitators for parents:
    1. attending the sessions? *[prompts: explore online vs in-person; explore with consideration of different characteristics]*
    2. engaging fully with the content/getting the most out of the sessions (e.g. making spoken contributions)? *[prompts: explore online vs in-person; explore with consideration of different characteristics]*
11. Do you think it is more helpful for parents to attend with or without their babies? Why?
12. Do you think it’s important to have any in-person sessions? Why? When?
13. Do you think other ‘parenting partners’ should be involved with the programme? *[prompts: in what way? would you have any concerns? how may these be overcome?]*
14. In your view, would it be helpful to implement this programme in specialist perinatal mental health services in England?
    1. What considerations would be needed?
    2. How would these challenges be best overcome?

Recap and thank for time.
